# Plasmonics-based detection of H_2_ and CO: discrimination between reducing gases facilitated by material control

**DOI:** 10.3762/bjnano.3.81

**Published:** 2012-10-31

**Authors:** Gnanaprakash Dharmalingam, Nicholas A Joy, Benjamin Grisafe, Michael A Carpenter

**Affiliations:** 1 College of Nanoscale science and Engineering, University at Albany-State University of New York, 257 Fuller Road, Albany, New York 12203, United States

**Keywords:** hydrogen detection, nanocomposites gold nanoparticles, optical sensor, plasmonics, physical vapor deposition, surface plasmon resonance

## Abstract

Monitoring emissions in high-temperature-combustion applications is very important for regulating the discharge of gases such as NO_2_ and CO as well as unburnt fuel into the environment. This work reports the detection of H_2_ and CO gases by employing a metal–metal oxide nanocomposite (gold–yttria stabilized zirconia (Au–YSZ)) film fabricated through layer-by-layer physical vapor deposition (PVD). The change in the peak position of the localized surface plasmon resonance (LSPR) was monitored as a function of time and gas concentration. The responses of the films were preferential towards H_2_, as observed from the results of exposing the films to the gases at temperatures of 500 °C in a background of dry air. Characterization of the samples by XRD and SEM enabled the correlation of material properties with the differences in the CO- and H_2_-induced LSPR peak shifts, including the relative desensitization towards NO_2_. Sensing characteristics of films with varying support thicknesses and metal-particle diameters have been studied, and the results are presented. A comparison has been made to films fabricated through co-sputtered PVD, and the calibration curves of the sensing response show a preferential response towards H_2_. The distinction between H_2_ and CO responses is also seen through the use of principal-component analysis (PCA). Such material arrangements, which can be tuned for their selectivity by changing certain parameters such as particle size, support thickness, etc., have direct applications within optical chemical sensors for turbine engines, solid-oxide fuel cells, and other high-temperature applications.

## Introduction

Sensors based on surface plasmon resonance have been a principal area of research in optical sensing devices [1–4]. The catalytic activity of highly dispersed gold particles either supported on metal oxides or embedded in metal oxides as discovered by Haruta et al. [5] served as pioneering work in the field of noble-metal catalysis in general, and particularly for plasmonics-based gas sensing. The extremely high sensitivity of the plasmon resonance peak to changes in the free-electron density of gold nanoparticles or a change in the dielectric function of the metal-oxide host material due to adsorbate reactions on surfaces makes this a viable chemical sensing technique. Although many metals, such as Cu, Al, and Ni [6], show characteristic plasmon peaks, they typically exhibit resonances at higher frequencies, which necessitates the use of complicated and expensive light sources. The use of gold or silver as the active sensing material does not warrant this, as the resonance wavelength region is in the visible and lower UV range, enabling the use of compact and inexpensive light sources. The choice of gold has thus been validated by its stability at high temperatures (the melting point of an unsupported 6 nm diameter Au nanoparticle, for example, is around 1150 K and decreases with decreasing particle size [7]).

There have been studies investigating the use of catalytically active gold or silver nanoparticles as optical sensors [8–12], along with theoretical models of the sensing response [13] and calculations of the sensitivity of the response to parameters such as shape, size and composition of the nanoparticles [14]. Ando et al. have reported, in one of the earlier investigations of sensing at high temperatures, the plasmonic sensing characteristics of Au nanoparticles when embedded in a CuO matrix, at a working temperature of 300 °C [15].

For consistent and sensitive detection of H_2_, CO and NO_2_, Rogers et al. and Sirinakis et al. used Au–yttria stabilized zirconia (Au–YSZ) films and reported sensing observations through hundreds of hours of laboratory testing between 500 and 800 °C [16–18]. While detection of these gases at high temperatures has been demonstrated, selectivity between these gases remains a challenging task, as many interactions between the different analyte gases and the film surface can be manifested as a change in the position of the plasmon peak. Selective detection of gases can be addressed either through a materials-development approach and/or the implementation of specific methods for data analysis. One example of this is selective chemiresistive sensor measurements with Ga_2_O_3_ materials. These studies showed that selectivity was enabled through the morphological tailoring of Ga_2_O_3_ and the use of both temperature changes as well as physical and chemical filters [19]. Another example in the direction of materials development is the work by Buso et al., who monitored specific wavelengths of the absorption spectrum of SiO_2_ sol–gel films containing NiO and Au NPs during gas exposures. They demonstrated the selective detection of H_2_ over CO, based on the differing response characteristics of the films in the different wavelength regions [20]. In another study, Gaspera et al. investigated the role of sol–gel-synthesized metal-oxide (NiO and TiO_2_) films that were coated over Au NPs. One of the motivations of this work was to examine if the catalytic activity of the sol–gel-coated Au NPs increased due to the reduction in temperature-driven sintering of the Au NPs by the metal-oxide films, which would serve to reduce the Au NP size. They showed that such an arrangement had a reversible response to ethanol [21]. In the direction of investigating the use of both materials and statistical algorithms to discriminate the different responses of a single film towards the CO, H_2_ and NO_2_ target gases, Joy et al. recently demonstrated a method of extracting spectral information from sensing experiments using both supervised and unsupervised statistical algorithms, linear-discriminant analysis (LDA) and principal-component analysis (PCA), respectively [22]. This study has practical benefits in that relevant wavelength regions can be identified from the entire plasmon spectrum, as determined by statistical algorithms that show the greatest selective detection of the target analytes.

In the current work, a Au–YSZ film has been fabricated through a layer-by-layer physical vapor deposition (PVD) procedure, and the response of the film to H_2_, CO and NO_2_ at 500 °C has been monitored by observing the change in the position of the localized surface plasmon resonance (LSPR) peak. This work employs a layer-by-layer approach, meaning that the Au was first deposited and annealed to form nanoparticles and was then followed by the deposition and annealing of the YSZ capping layer. The metal-oxide overcoat has a crucial role in restricting the growth of the Au NPs during long-term high-temperature exposures, and its thickness has a direct impact on the number of oxygen vacancies in the film. The vacancies are introduced into the film through the yttria dopant in zirconia. YSZ is an excellent oxygen-ion conductor at temperatures greater than 300 °C, with almost 99% of its conductivity being due to the transport of oxygen ions above this temperature [23]. In the current study, an investigation into the dependence of the chemical sensing on Au particle size coupled with the YSZ-overcoat thickness has been performed for the first time. The resulting material properties of these films have produced a unique sensing dependence, which has enabled an enhanced detection of H_2_ by a factor of 4 in comparison to CO. Such a strong difference in the detection of these two reducing gases is significant with respect to meeting the challenge towards selectivity. An additional analysis that exemplifies the differences between the two most prominent films was carried out by using PCA.

## Results and Discussion

### Exposure conditions and sensing results

**Figure 1:**
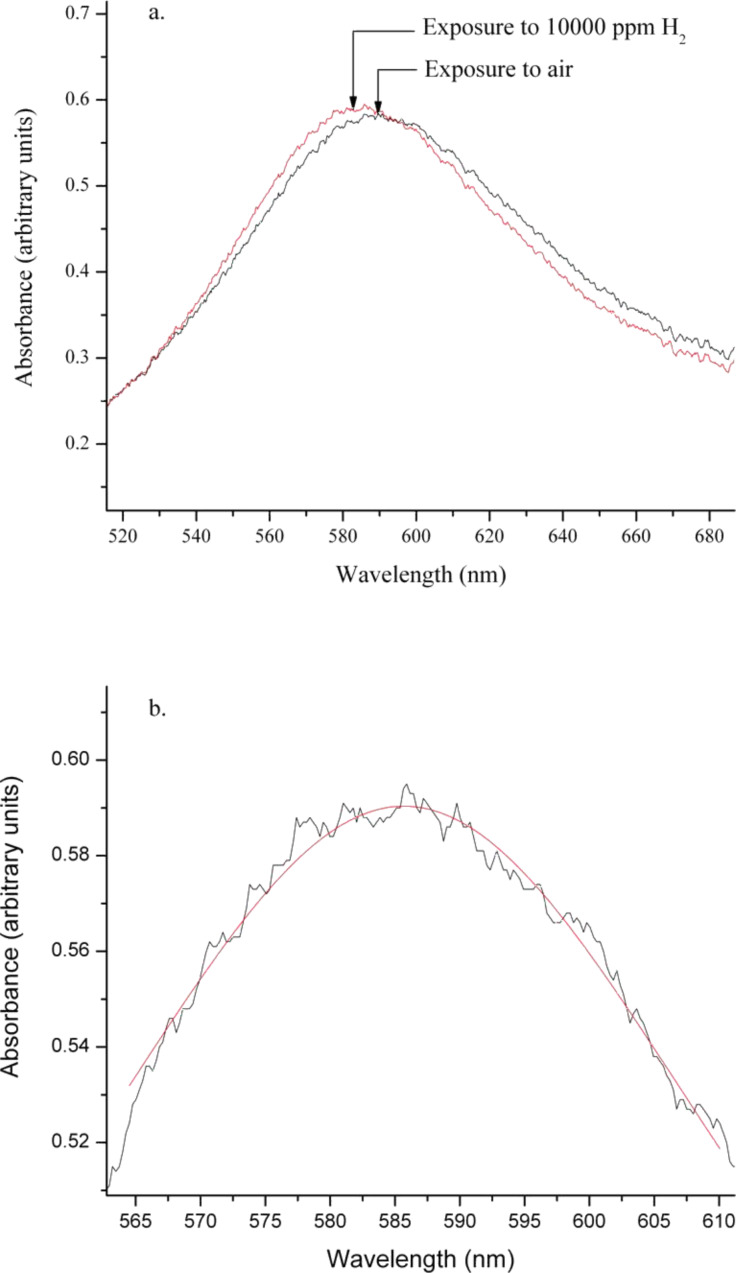
**(**a) Change in peak position of the small-particle sample between exposures to air and H_2_. The lower plot (b) shows a sample Lorentzian fit (red line) of the absorbance spectrum to the spectrometer data (black line), which was used to monitor the change in peak position.

**Figure 2:**
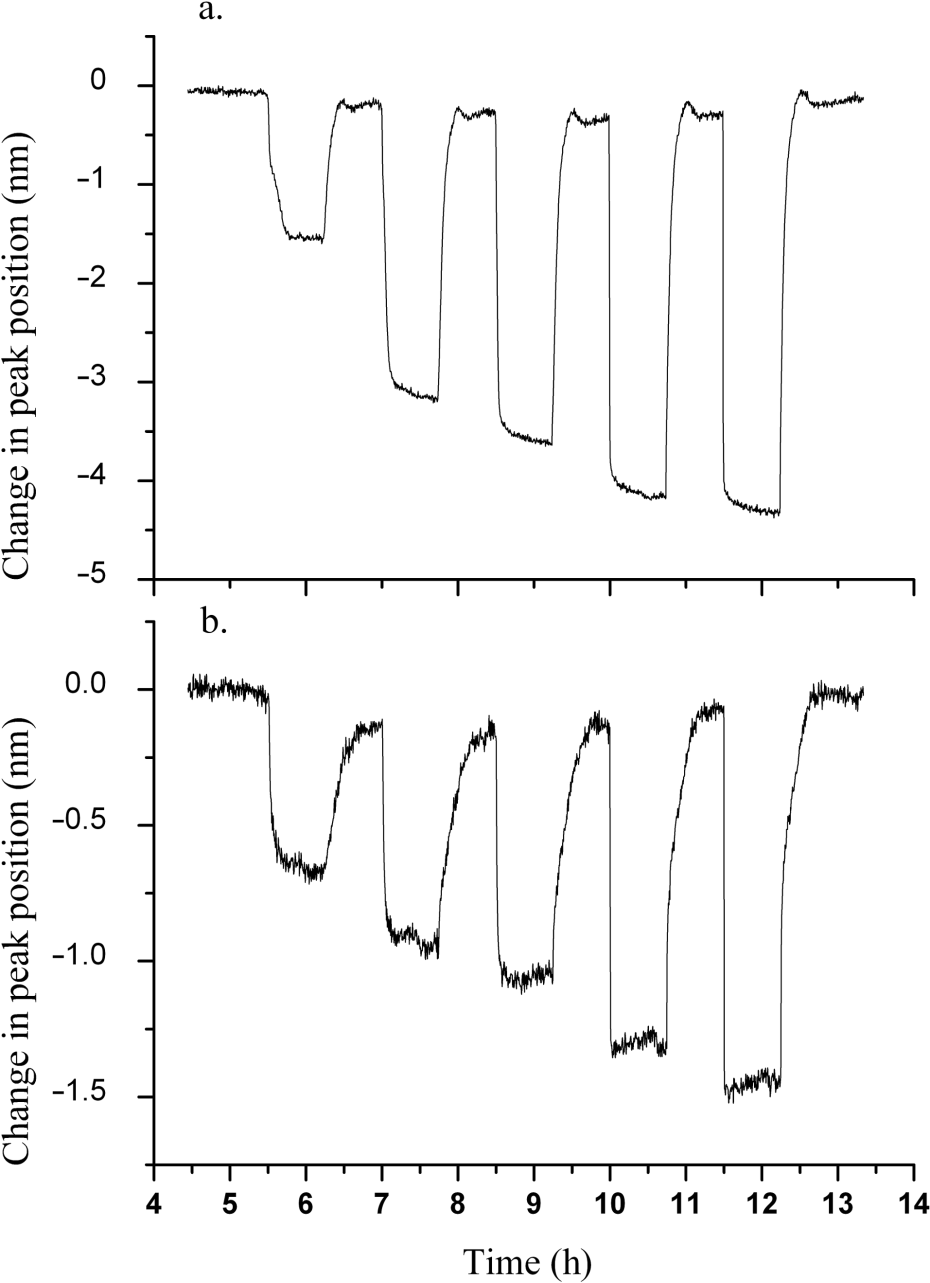
Hydrogen-exposure plots of (a) small-particle and (b) medium-particle samples. Concentrations of 200, 500, 1000, 5000 and 10000 ppm of H_2_ in an air background were tested and are overlaid for the two samples, corresponding to increases along the time axis.

**Figure 3:**
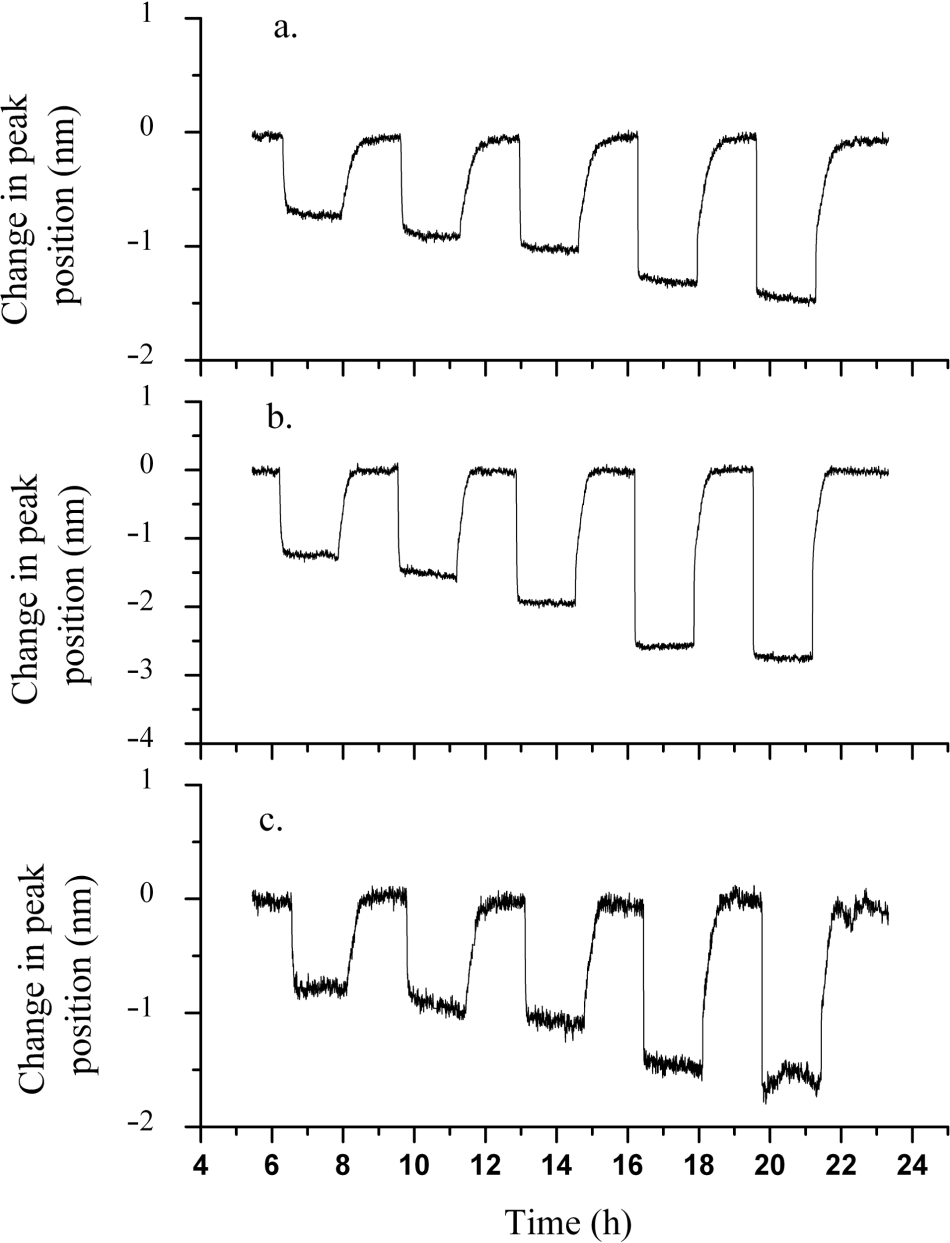
Hydrogen exposure plots of (a) thinner gold, (b) co-sputtered and (c) large-particle samples. The exposures are overlaid and correspond to concentrations of 200, 500, 1000, 5000 and 10000 ppm of H_2_ in air, respectively, increasing along the time axis.

The gases tested were H_2_, CO and NO_2_ in an air background. The exposure concentrations were 200, 500, 1000, 5000 and 10000 ppm for H_2_ in dry air; 20, 50, 100, 500 and 1000 ppm for CO in dry air; and 2, 5, 10, 50 and 100 ppm for NO_2_ in dry air. For ease of discussion, all samples with 3 nm Au but with 5/10/20 nm YSZ are referred to as medium-, large- and small-particle samples, respectively, and the film with 1.5 nm Au and 20 nm YSZ is referred to as the thinner gold sample. For comparison of the sensing data, a film fabricated by co-sputtering of the Au and YSZ, which was previously studied, was selected and is referred to as the co-sputtered sample. The exposure temperature was 500 °C, and all samples were allowed a warm-up time of five hours before the first exposure. An example of the shift in peak position, in this case for the small-particle sample on exposure to H_2_, is shown in Figure 1 along with a sample Lorentzian fit used to determine the LSPR peak position, which was used as the sensing signal and monitored as a function of time. As these H_2_ exposures were repeated a total of three times in a 72-hour experiment, a subset of the results for the hydrogen exposures are shown in Figure 2 and Figure 3.

From the exposure plots the most obvious observation is the shift in the plasmon resonance peak towards shorter wavelengths upon exposure to H_2_. This shift is likely the result of interfacial charge-transfer reactions between H_2_ and the oxygen anions forming water as the product [12–14]. As a result, electrons are transferred to the Au NPs inducing a blue shift or increase in LSPR frequency, ω, as characterized by the Drude model in Equation 1.

[1]
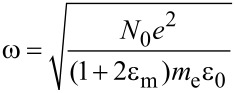


In the above equation *N*_0_ is the free-electron density of the Au particle, *e* the electron charge, ε_m_ the dielectric constant of the matrix and ε_0_ the permittivity of vacuum [24]. These reactions will also likely induce a change in the polarizability of the YSZ matrix, changing the dielectric constant. The shift in the plasmon peak position will therefore be a result of the combined effect of the charge exchange and the change in dielectric properties of the YSZ. Other chemical reactions between H_2_ and YSZ could also induce a change in the dielectric function, and while the adsorption of hydrogen is an activated process, the activation energy is typically less than 1 eV [25]. The uptake of hydrogen as an OH species by a zirconia matrix at temperatures between 673 and 873 K has been confirmed through studies using infra-red spectroscopy [26]. A significant difference between this previous study and practical studies of emission-gas sensing is that the measurements were not done in the presence of background oxygen. However, if H_2_ were to react in the presence of an oxygen background, (such as in air) these reactions would induce a change in the dielectric function of the matrix. Such operando studies, combining chemical sensing measurements with analytical methods that simultaneously probe the reaction mechanism that induces the sensing response, remain a challenging experiment under relevant atmospheric sensing conditions.

**Figure 4:**
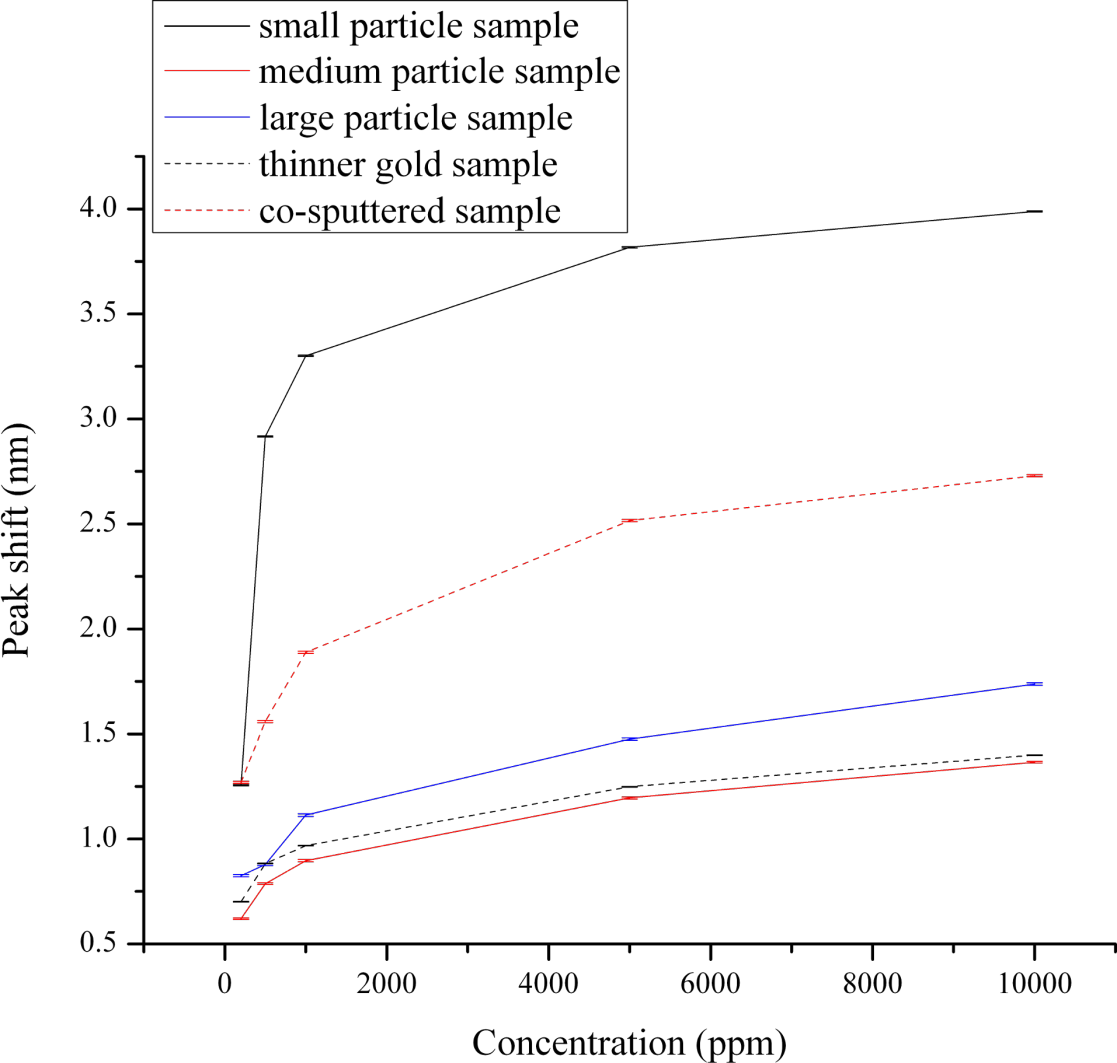
Calibration curves of all investigated samples for hydrogen. Error bars for each of the five separate H_2_ exposures have been included.

Inspection of Figure 2 and Figure 3 shows that while each of the five films responds well to H_2_ as evidenced by the significant shifts in the plasmon peak position and the stable baseline peak position during the air cycles, the small-particle film is the most responsive among all samples. This is evident from the maximum change in the plasmon peak position as a function of H_2_ concentration and is more clearly shown in the calibration curves in Figure 4. The data plotted is the change in LSPR peak position as a function of the H_2_ concentration, with the values of the LSPR peak position representing the weighted average of three repeats for each gas concentration, and the error bars representing the uncertainty in the weighted average. The enhanced response of the small-particle film towards H_2_ is quite interesting and is approximately a factor of 1.5 better than the co-sputtered film that was used in previous studies. The difference in response towards H_2_ as a function of the film composition used in Figure 2 and Figure 3 requires some further comparison with respect to their morphological differences. One determining factor may be the respective oxygen vacancies in each of the films. For the calculation of the oxygen-vacancy concentration in all films, a general assumption was that the YSZ film had a cubic fluorite lattice structure. The calculations were performed by taking into account the deposited area, thickness and lattice parameter of the YSZ film, and the number of oxygen vacancies that would be formed due to the yttria dopant level.

Calculation of the number of oxygen vacancies per square centimeter led to the following numbers for the films: 8.16 × 10^15^/cm^2^ for the small-particle sample and the thinner gold sample, 2.04 × 10^15^/cm^2^ for the medium-particle sample and 4.08 × 10^15^/cm^2^ for the large-particle sample. Given the proposed reaction mechanism, the enhanced response towards H_2_ for the small-particle sample may be due to the highest oxygen vacancy concentration in this film among all the samples, which would facilitate an increase in H_2_ adsorption, coupled with the smaller-diameter Au particles. Although the thinner Au sample has the same thickness of YSZ and hence essentially the same number of oxygen vacancies as the small-particle sample, the smaller Au particles in the latter case lead to an increased response. This may be attributed to the number of adsorption sites (such as defects, etc., which are the preferred sites for adsorption [27]) being higher for this sample. For the medium- and large-particle samples, the reduced response to H_2_ in comparison to the small-particle sample may be a direct consequence of the fact that the reduced YSZ thickness would reduce the number of oxygen vacancies, decreasing the number of oxygen anion species for reaction. In comparing these films, it is noted that while there has not been a direct scaling of the magnitude of the change in plasmon peak position with YSZ thickness, the general qualitative trend of an increased response with an increase in the number of oxygen vacancies appears to be followed.

**Figure 5:**
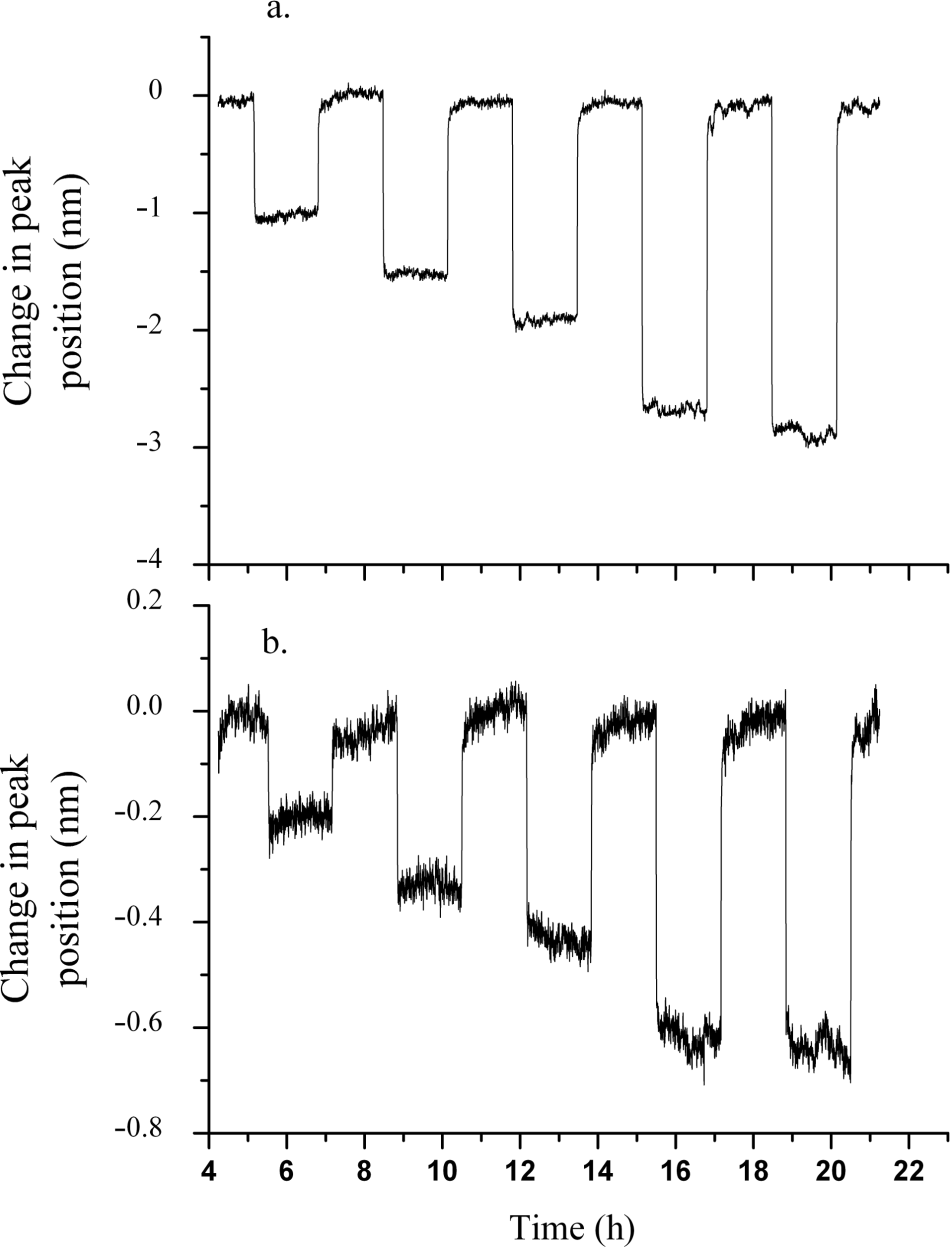
Exposure plots of (a) co-sputtered sample and (b) small-particle sample to CO. The concentrations tested were 20, 50, 100, 500 and 1000 ppm, respectively, in the increasing direction of the time axis.

The samples were also tested for their response towards CO, and a subset of the exposure results are shown in Figure 5.

**Figure 6:**
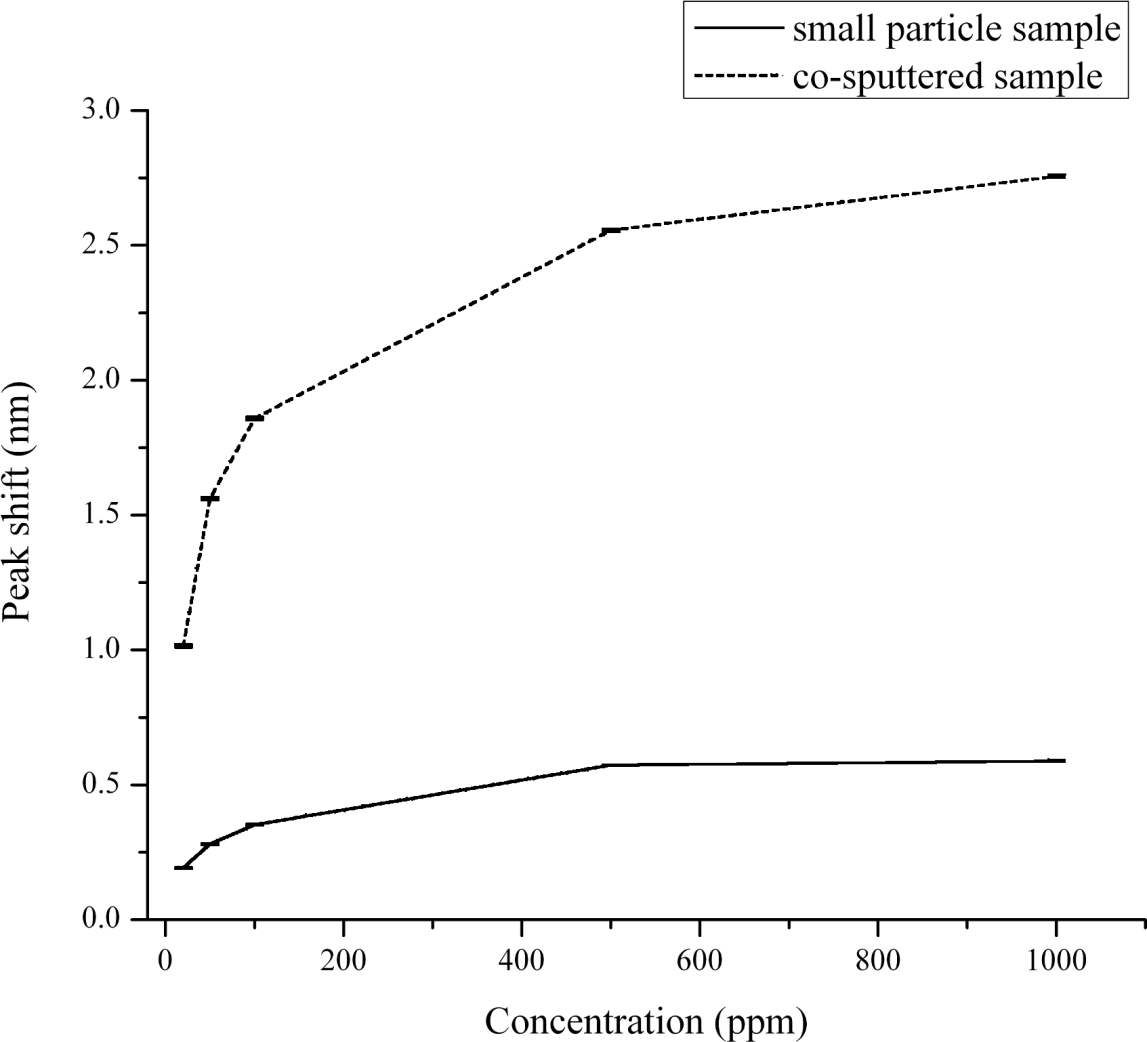
CO Calibration curves for the small particle sample and the co-sputtered sample. The error bars are shown for all concentrations.

Similar to H_2_, the mechanism of the LSPR shift can be explained as follows. The adsorption of CO leads to the extraction of oxygen ions from the lattice, followed by injection of electrons from the O^2−^ ions into the matrix and CO_2_ desorption. This can increase the free-electron density of the Au particles and cause the shift of the plasmon peak to the lower wavelength region of the spectrum. Similar to the H_2_ experiments, these reactions could also affect the dielectric function. Although all samples were investigated for their response to CO, only the co-sputtered and small-particle samples showed a detectable response for all CO concentrations, as shown in Figure 5. The sample with the highest response was the co-sputtered sample with the small-particle sample having a small but measurable change in LSPR peak position. Each of the other samples either had a very small peak-shift response to the higher concentrations of CO, or none that was detectable above the baseline noise. The catalytic reaction of CO to CO_2_ has been found to have a strong dependence on the Au NP size. Specifically, for inert metal-oxide supports, an enhancement in CO adsorption on the surface occurs only for particles with diameters less than 2 nm [28]. However, activity towards CO oxidation also occurs for particle diameters ranging from 12 to 30 nm when the particles are supported on active metal-oxide supports, such as Fe_2_O_3_ and YSZ. These supports are able to trap oxygen due to the presence of oxygen vacancies in their lattice. The combined effect of dissociative adsorption of oxygen on these supports with the activity of the Au nanoparticles produces an active material towards CO oxidation [29]. Thus, it was proposed that the ability of the YSZ support to provide reactive oxygen for CO oxidation increases the critical diameter for CO oxidation enhancement into the 10–30 nm range. It was noted, however, that the catalytic activity does decrease with increasing particle diameters, even for active supports. Rogers et al. [30] have also discussed the enhancement in sensitivity with decreasing particle sizes of Au for the detection of H_2_, NO_2_ and CO gases in a background gas containing mixtures of N_2_ and O_2_ as well as air. Thus, we propose that the reduced particle size, in addition to the fact that the metal oxide used in this study serves as an active support, is the reason for the observed increase in response of the co-sputtered sample (which has a mean particle size of 13 nm) when compared to the other samples (the sample with the smallest particles having a mean diameter of 48 nm). The calibration curves for CO response for the two samples (co-sputtered and small-particle sample) are shown in Figure 6.

**Figure 7:**
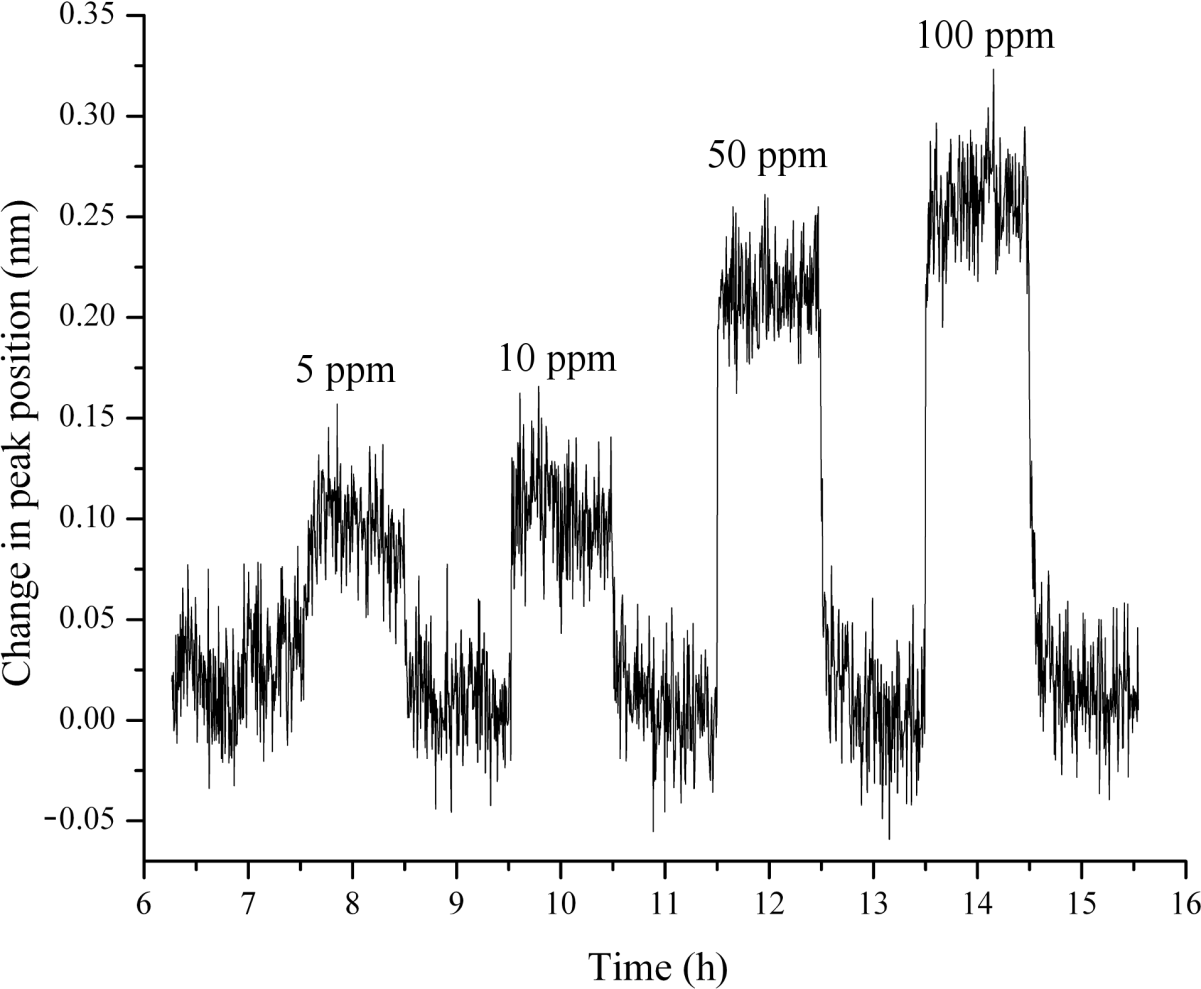
Exposure plot of small-particle sample to NO_2_. The concentrations tested were 2, 5, 10, 50 and 100 ppm, and only the last four concentrations, for which the sample had a detectable response, have been shown here, in order of increasing time.

Exposure to NO_2_ should cause a red shift in the plasmon peak position, as it is known that NO_2_ dissociates on Au–metal-oxide composites [31], and, provided oxygen vacancies are available, the dissociated oxygen would be adsorbed as either surface or lattice oxygen anions, O^−^ or O^2−^ respectively. This would cause a reduction in the free-electron density of Au and would decrease the plasmon frequency, causing the aforementioned red shift. Interestingly, all the layer-by-layer samples were relatively desensitized to NO_2_. This conclusion was drawn from the fact that the maximum peak shift for the highest concentration of NO_2_ was approximately 0.25 nm, a factor of 2 lower than the CO response, which in itself was much lower than the response towards H_2_. The reason for the low response is likely the unavailability of oxygen vacancies in the YSZ matrix, as the samples are exposed to a constant air background. Thus, an almost completely saturated matrix (i.e., the vacancies are saturated due to O^2−^ formation from dissociative adsorption of O_2_) is hypothesized as the cause for the low response of the samples towards NO_2_. This hypothesis is further supported by the work of Rogers et al. [17], wherein the response to NO_2_ was found to increase when the concentration of O_2_ in the background gas was reduced from 20% to 5%. The exposure plot of the small-particle sample, which had the highest response, is shown in Figure 7 for NO_2_ exposures of 5, 10, 50 and 100 ppm.

To summarize, the response of the small-particle sample to H_2_ was found to be higher than that of the co-sputtered sample (a 150% increase in response, to 10,000 ppm of H_2_), while the response to CO is much lower (a 450% reduction in response, to 1000 ppm of CO). Such a varied response to two reducing gases raises the possibility for the employment of these samples in a sensing array for the selective detection of H_2_ and CO. We are currently probing the optimization of the small-particle-sample configuration so that it will have an increased response to H_2_, while being even more desensitized to CO and NO_2_. Optimization of the particle sizes and the thickness and chemistry of the metal-oxide support may help realize these objectives. To elicit the selective response of the small-particle sample towards CO and H_2_, PCA was carried out on datasets for both the small-particle sample and the co-sputtered sample; the co-sputtered sample being selected because of its almost identical response towards H_2_ and CO in terms of its respective change in plasmon peak position upon gas exposure.

### Principal component analysis

The purpose of performing PCA is to extract as much information as possible from the absorbance spectrum of a sample in order to observe a unique response for each of the analytes. Of the many multivariate methods available, PCA is attractive due to its simplicity of application [22,32–33]. It is an unsupervised technique that reduces the dimensionality of a dataset while still retaining as much variance in the data as possible. In order to do this, data points are transformed onto a new set of orthogonal axes that run in directions of maximum variance in the data. Dimensionality can be reduced by projecting the data points onto a subspace of just the first two principal components (PCs), which typically retain most of the variance in the data.

**Figure 8:**
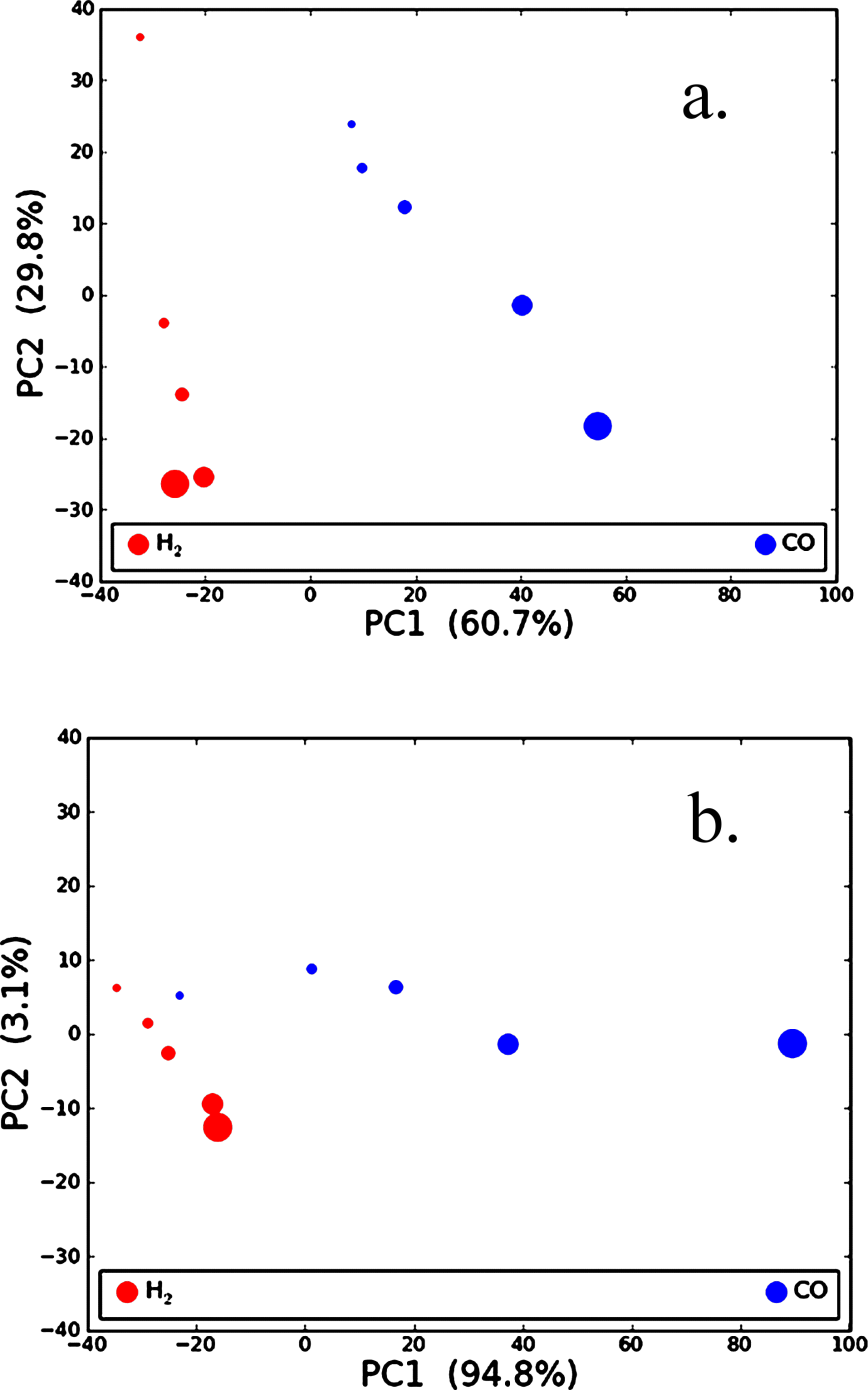
PC scores plot for (a) small-particle sample and (b) co-sputtered sample in an air background at 500 °C. The percent variance in the data described by each PC is listed in parentheses and data marker size increases with analyte concentration. The degree of separation between the H_2_ (red) and CO (blue) markers is a qualitative measure of the difference in response of the sample to the two gases.

For the present analysis, the observations, or data points, consist of each of the H_2_ and CO concentrations (five each for H_2_ and CO). The measured variables consist of 1570 individual wavelengths in the absorption spectrum between 450 and 850 nm. PCA was performed with the Python programming language by using singular value decomposition (SVD). Details on the mathematical procedure can be found elsewhere [34–35]. The observations were then projected onto the principal component 1 (PC1) and principal component 2 (PC2) axes and plotted in Figure 8, which are known as the PC scores plots.

The PC scores plots can be viewed qualitatively based on the separation between the clusters of points representing H_2_ exposure and clusters representing CO exposure. As the separation improves, it becomes easier to distinguish one analyte from the other. Given the range of concentrations tested for this experiment, points tend to form lines rather than clusters. However, a distinct separation between the H_2_ and CO data points is seen for both samples in Figure 8. Although the analysis is limited in terms of the number of observations (i.e., data points), the results indicate that the small-particle sample has a distinction between H_2_ and CO at all concentrations, while the co-sputtered sample has less separation of the data points at the lower concentrations. This suggests that the small-particle sample appears to be better suited to classifying the analytes at the lower concentrations. This observation is quite important as the selective detection of CO and H_2_ for many types of metal-oxide-based sensing applications is problematic since they both react readily with the oxygen anion species and produce a similar response on the transducer of interest [1]. The results from this current study show that by tuning the material properties, a single metal-oxide film can have a CO response that is a factor of about 5 lower than that of H_2_, evident from a plasmon peak shift of 0.6 nm for 1000 ppm of CO as opposed to 3.3 nm for 1000 ppm of H_2_.

## Conclusion

In this work, we have demonstrated that morphological modification of a Au-YSZ nanocomposite results in a vivid change in the response to H_2_ and CO gases, and also an apparent desensitization to NO_2_. The morphological switching mentioned here results from a change in the sample fabrication method from a co-sputtered Au–YSZ film, in which the Au particles are embedded in the YSZ matrix, to a layer-by-layer process for nanocomposite preparation. PCA analysis was employed to illustrate the difference in response between H_2_ and CO for the co-sputtered and small-particle sample. These composites could serve as potential sensing materials in a sensor array for selective detection of H_2_, CO and NO_2_. Experiments are currently underway to determine the optimal configurations of these samples for a selective response to all gases. Further investigations to describe quantitatively and qualitatively the mechanism of charge exchange in terms of the reaction kinetics are in progress. Additional work to investigate the adsorbed species during the gas exposures through Raman spectroscopic characterization is on track, such that a predictive method of optimal sample preparation and configuration can be applied in the future.

## Experimental

### Film fabrication

Radio-frequency co-magnetron confocal physical-vapor deposition was used in the synthesis of all films. The general fabrication procedure was as follows: (i) deposition of 1.5 or 3 nm Au on quartz substrates with half of the substrate masked. The masked region allows for a reference spectrum to be continuously recorded during the spectral measurements of the Au–YSZ nanocomposite film. (ii) Annealing of the deposited Au film at a temperature of 900 °C for five minutes in an Ar environment with a flow of 2000 sccm. This annealing step results in the transition of the Au film to Au NPs. (iii) Deposition of the YSZ capping layer (5/10/20 nm thickness depending on the samples) on the Au film. And finally, (iv) annealing of the deposited films for three hours at 800 °C in a 2000 sccm flow of Ar to stabilize them for the sensing experiments. A half hour ramp-up and a final cool-down of the samples in argon were part of the annealing process.

The PVD targets used were Au of 99.99% purity and YSZ (99.9% purity) with a 5 wt % doping of yttria. The use of a constant annealing time and temperature for each of the samples lead to samples with Au particle sizes resulting from a change in PVD deposition conditions. The trends in particle sizes were corroborated by using environmental scanning electron microscopy (ESEM) and X-ray diffraction (XRD) The co-sputtered sample was fabricated by co-sputtering of Au and YSZ, by using a procedure described elsewhere [22]. Table 1 lists the sample nomenclature and the PVD deposition parameters. The selection of the co-sputtered film was based on the fact that the response to CO of the co-sputtered film was relatively similar in magnitude to its response to H_2_. This is in stark contrast to the unique sensing response observed for films deposited in a layer-by-layer fashion. The layer-by-layer process has enabled the use of a unique set of samples with varying Au NP size and Au atomic percentage. Specifically, calculations of the Au content in each of the films revealed values of 2%, 8%, 4% and 1% for the small, medium, large and thinner gold samples, respectively, with the balance of these films being YSZ. The co-sputtered film was shown to have 9 atom % Au.

**Table 1:** Sample nomenclature and physical-vapor-deposition parameters.

Sample ID	Thickness of Au layer (nm)	Thickness of YSZ overcoat (nm)	Deposition rate of Au layer (Å/s)	Deposition rate of YSZ overcoat (Å/s)

Small-particle sample	3	20	0.3	0.3
Medium-particle sample	3	5	0.3	0.3
Large-particle sample	3	10	0.3	1.4
Thinner-gold sample	1.5	20	0.3	1.4

### Optical sensing apparatus

**Figure 9:**
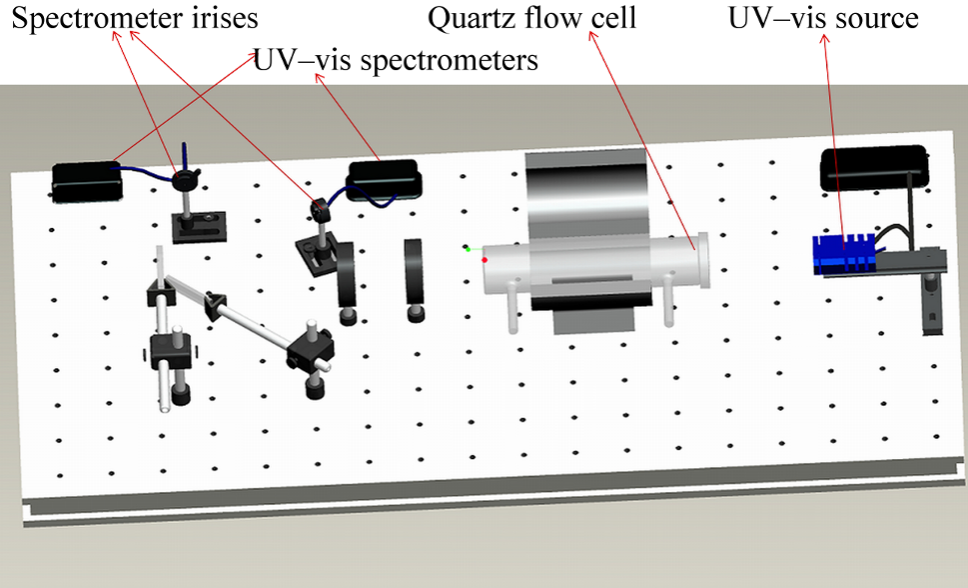
Optical sensing apparatus. From left: UV–vis source, quartz flow cell with gas inlet and outlet ports, tube furnace, focusing lenses, beam splitters and spectrometers.

The sensing apparatus used for the experiments is shown in Figure 9. The setup consists of, from right to left, an Ocean Optics tungsten halogen source with an emission wavelength range of 360–2500 nm; the quartz flow cell in which the sample is placed in the optical centerline by mounting in a Macor holder; a tube furnace for temperature control up to 900 °C; two lenses to create the optical image of the sample; and two beam splitters, which direct the beam onto two Ocean Optics spectrometers, one for recording the reference spectrum and the other for monitoring the spectrum from the sample. This setup is a simpler and lower-cost alternative to the 2-D CCD-imaging-based optical apparatus used previously [22]. The gas flow was regulated by computer-controlled mass-flow controllers supplied by MKS, and the total flow rate was maintained constant at 2000 sccm for all exposures.

### Sample characterization

For characterizing the deposited samples, ESEM and XRD were used. ESEM analysis was carried out by using a FEI E-SEM 600, and the crystallite sizes from the ESEM images were calculated using ImageJ software, assuming spherical particles of gold. The particle (crystallite) diameters for all the samples are tabulated in Table 2.

**Table 2:** ESEM characterization results for PVD prepared samples. All values are in nanometers.

Sample ID	Mean crystallite size	Standard deviation in crystallite size

Small-particle sample	48	14
Medium-particle sample	64	23
Large-particle sample	125	48
Thinner gold sample	57	16

**Figure 10:**
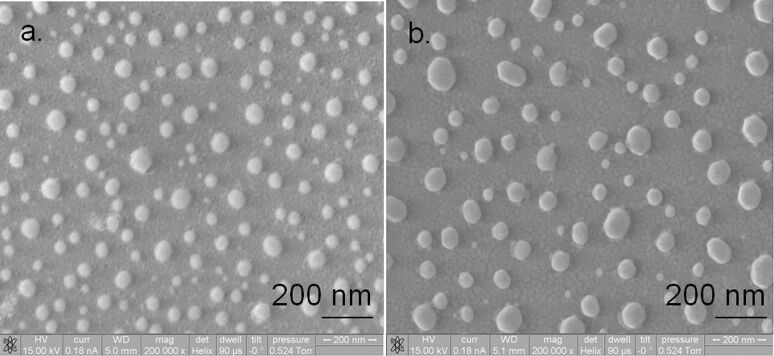
ESEM images of (a) small-particle and (b) medium-particle films. The annealing times were kept constant for all films so that variations in the particle size would be entirely a consequence of the deposition parameters. Diameter measurements were done on 172 particles for (a) and 89 particles for (b) to get the average sizes.

**Figure 11:**
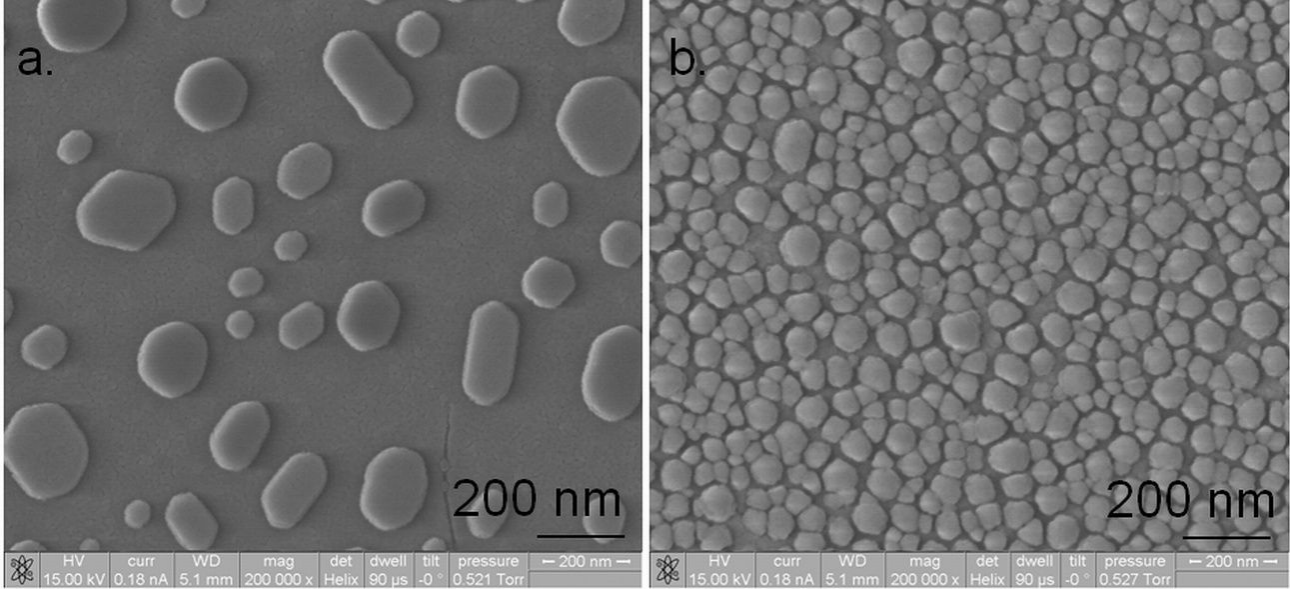
ESEM images of (a) large-particle sample and (b) thinner gold sample. Diameter measurements were carried out on 34 particles for (a) and 545 particles for (b) to arrive at the average sizes.

Figure 10a,b and Figure 11a,b show ESEM images of the small-, medium- and large-particle samples, and the thinner gold sample, respectively. From the ESEM images the Au crystallites can be clearly seen, and the scaling of the Au particle size with decreasing YSZ thickness is obvious, except for the fact that the particle size of the large-particle film should have been smaller than that of the medium-particle film due to the larger YSZ thickness. We speculate that this deviation is due to the fact that the deposition rate during the sample preparation of the former film was three times higher than the latter, thereby possibly changing the morphology of the YSZ film and allowing an increased sintering of the Au crystallites during annealing.

XRD analyses were performed on the samples with a Scintag XDS 2000 by using Cu Kα radiation (wavelength of 1.54 Å). The crystallite sizes were determined by using the Scherrer Equation 2,

[2]
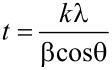


Where *t* is the crystallite size in nanometers, *k* is the Scherrer constant (assumed to be 0.9), λ is the wavelength of the radiation, β is the value of the full width at half maximum (FWHM) of the Gaussian fit to the XRD peak profile, and θ is the diffraction angle. The tabulated values of the crystallite sizes for all samples have been included in Table 3, along with the calculated values from ESEM data for comparison. The instrumental contribution to the peak width for the XRD was accounted for by using the XRD profile fit of a thick gold sample with large Au particles (greater than 500 nm in diameter) within a YSZ film that was 40 nm thick.

**Table 3:** Crystallite sizes calculated from XRD data and the Scherrer equation. ESEM particle sizes are shown for comparison. All values are in nanometers.

Sample ID	Average crystallite size from XRD	Average crystallite size from ESEM

Small-particle sample	58	48
Medium-particle sample	129	64
Large-particle sample	120	125
Thinner gold sample	63	70

The crystallite sizes from the ESEM and the XRD are mostly in reasonable agreement with respect to the average diameters. The small variations may be attributed to the fact that the calculations of the particle sizes for ESEM were not averaged over the entire sample surface, which could have resulted in a closer agreement with the XRD values. This is particularly true for the differences observed for the medium-particle sample, which shows values that are different by a factor of about 2.
